# Protective effect of high dose short term statin therapy with normal saline in prevention of contrast-induced nephropathy among iodixanol-receiving patients

**DOI:** 10.12861/jrip.2012.15

**Published:** 2012-01-01

**Authors:** Houshang Sanadgol, Siavosh Abdani, Peyman Tabatabaiee, Mehdi Mohammadi

**Affiliations:** ^1^Department of Internal Medicine, Zahedan University of Medical Sciences, Zahedan, Iran; ^2^Faculty of Health, Zahedan University of Medical Sciences, Zahedan, Iran

**Keywords:** Statin, Contrast-induced nephropathy, Renal failure

## Abstract

Contrast media agents are applied for various diagnostic imagines, however, contrast-induced nephropathy (CIN) limits its usage. Statins have been found to prevent CIN via various mechanisms. However, study regarding the beneficial property of simvastatin as a kind of statin is scarce. This study was aimed to evaluate the efficacy of high dose short term statin therapy against nephrotoxicity of iodixanol. 194 patients were divided equally to control and statin-treated groups. Control group (placebo) received normal satin before and after angiography and statin-treated patients received simvastatin (80 mg/day) plus normal saline before and after angiography. Simvastatin and normal saline were started 12 hours before to 12 hours after the procedure, and serum creatinine before and two consecutive days after procedure were assessed. Estimated glomerular filtration rate (eGFR) was calculated using modification of diet in renal disease (MDRD ) method. In the first 24 hours after procedure, there was no difference between two groups, however after 48 hours of treatment, a significant difference for eGFR between two groups with more values in statin treated group was observed (p=0,002). Prophylactic administration of statins along with hydration may be associated with less contrast-induced nephropathy.

**Implication for health policy/practice/research/medical:**

Prophylactic administration of statins along with hydration may be associated with protective effects against contrast-induced nephropathy.

## Introduction

Contrast media agents applied for various diagnostic imagines, however, contrast-induced nephropathy (CIN) limits its usage (1). CIN is a leading cause of acute renal injury and is associated with significant morbidity and mortality (1,2). Indeed, CIN is an acute deterioration of kidney function following administration of contrast media which is thought to be mediated by the increased production of reactive oxygen species (ROS) within the kidney (2-4). Recent findings suggested both direct and indirect effects on renal tubules including biochemical and hemodynamic disturbance such as, hypotension, medullary ischemia and prerenal dehydration (3,4). Impairment of kidney perfusion leads to hypoxic conditions resulting in acute tubular cells necrosis (1-5).

## Objectives

Various studies suggested beneficial effects of statins against CIN-induced renal toxicity, however study regarding the beneficial property of simvastatin as a kind of statin is scarce and therefore we aimed to study the efficacy of high dose short term statin therapy against nephrotoxicity of iodixanol.

## Patients and Methods

### Patients

The study was a prospective randomized placebo-controlled trial which was conducted from October 2010 through November 2011, in an educational Hospital of Zahedan University of Medical Sciences. Among all the patients referred for coronary angiograph with iodixanol, 248 patients firstly met the inclusion criteria and were enrolled in the study. However, thirty seven patients were excluded due to prior statins consumption within 7 days of study and/or receiving intravenous fluid therapy within past 48 h.

Two hundred eleven patients were randomized to simvastatin and placebo using table of random numbers (102 simvastatin and 109 placebo groups). Patients in simvastatin received 80 mg simvastatin daily (total dose of 160 mg), started 48 hours prior the coronary angiography. Patients in placebo group received two sugar containing capsules daily alongside with the case group for 48 hours. All patients were hydrated with normal saline with the rate of 50 mL per hour from 12 hours before to 12 hours after the procedure.

### Laboratory methods

In each group, serum creatinine was measured once before administration of simvastatin as baseline and 2 days after the procedure using autoanalyzer. Estimated glumerular filtration rate (eGFR) was measured with modification of diet in renal disease (MDRD) formula. Any alteration in GFR was statistically analyzed in both groups in days 2 and 3 after coronary angiography. The angiography was done based on the routine procedure through femoral vein, using Iodixanol as the contrast media. The effect of hypertension, diabetes, chronic kidney disease, age and gender on CIN was also assessed.

### Ethical issues

(1) The research followed the tenets of the Declaration of Helsinki; (2) informed consent was obtained; (3) the research was approved by ethical committee of Zahedan University of Medical Sciences‏.

### Statistical analysis

Data were analyzed using *t*-test and paired * t-*test between both groups. The effect of simvastatin on contrast nephropathy was evaluated based on any statically significant change in GFR. P<0.05 was considered significant.

## Results

Of two hundred eleven patients (102 simvastatin and 109 placebo), four patients in placebo group and 13 patients in simvastatin group did not cooperate and were excluded resulting in 96 patients in placebo group and 98 in simvastatin group. The baseline demographics of patients are demonstrated in Table 1. Statistically, there were no significant changes in first 24 h (p=0.190) after the angiography but changes after 48 h were significant. There was no significant difference between diabetic and non-diabetic patients of simvastatin group in post angiographic renal function‏ (p=0.25).‏ Other variables such as presence of hypertension, age & gender had also no influence on the effect of simvastatin on post angiographic eGFR (Table 1).

**Table 1:** Variables in simvastatin and placebo groups

Variable	N/S	Simvastatin	*P*
Age (y)	10‏±56	10‏±55	0.47
Male	50%	47 %	0.67
DM	28%	21%	0.25
HTN	30%	31%	0.87
GFR‏ _1_ ‏(cc/min)	83.6±37.5	72.9±25.8	0.02
GFR_2_ ‏(cc/min)	78.3±30.3	81.4±28.6	0.45
GFR_3_ ‏(cc/min)	74.9±25.4	79±23.5	0.24
GFR : Glomerular Filtration Rate, GFR_1_: Basal GFR, GFR_2_: GFR after 48 h, GFR_3_: GFR after 72 h			

## Discussion

This study showed that, during the first 24 hours after procedure, there was no difference, between two groups, however after 48 hours, a significant difference of eGFR between two groups with more values in statin receiving group was seen. Analysis of the data in control group declared that fluid therapy in first 24 h partially prevented decreased renal function but had no preventive effect after 48 h. CIN is an acute worsening of kidney function following administration of contrast media (1-3). CIN is associated with mortality, long-term morbidity and increased health care costs (2,4). Al-Otaibi *et al*. in a study on a group of rats found simvastatin had protective effects against CIN nephrotoxicity (2). In a study on 228 patients with acute coronary syndrome undergoing selective percutaneous coronary intervention who were randomly divided into simvastatin 20 mg group and simvastatin 80 mg group, Xinwei *et al*. found that in simvastatin 80 mg group the creatinine clearance recovered to baseline level at 48 hours, however it failed to do so in the simvastatin 20 mg group. The creatinine clearance was greater at 24 and 48 hours in the simvastatin 80 mg group than that in the simvastatin 20 mg group. They concluded that, pretreatment with simvastatin 80 mg before percutaneous coronary intervention could further decrease the occurrence of contrast-induced nephropathy compared with simvastatin 20 mg (6). It is evident that, statins have anti-inflammatory properties that are not directly related to their cholesterol-lowering efficacy (1-4). It is possible that, this drug by prevention of lipid peroxidation and tissue fibrosis, suppression of neutrophil infiltration and preservation of antioxidant glutathione protects kidneys (2-4).

## Conclusion

Prophylactic administration of statins along with hydration may be associated with less contrast-induced nephropathy.

## Authors’ Contributions

HS and SA defined the aims of research. SA, PT and MM prepared the paper. HS edited the final manuscript.

## Conflict of interests

The authors declare that they have no conflict of interest.

## Ethical considerations

Ethical issues (including plagiarism, data fabrication, double publication) have been completely observed by the author.

## Funding/Support

This study was supported by a grant from Zahedan University of Medical Sciences.
